# Effect of Cross-sectional Designs on Torsional Resistance of Endodontic Nickel–Titanium Files: A Finite Element Study

**DOI:** 10.1055/s-0044-1791785

**Published:** 2024-11-07

**Authors:** Natchaphon Nanthaprathip, Sarita Morakul, Sirawut Hiran-us, Pairod Singhatanadgid

**Affiliations:** 1 Department of Mechanical Engineering, Faculty of Engineering, Chulalongkorn University, Phyathai, Patumwan, Bangkok, Thailand; 2 Department of Operative Dentistry, Faculty of Dentistry, Chulalongkorn University, Bangkok, Thailand

**Keywords:** torsional resistance, shear stress, endodontic, rotary file, cross-sectional design, finite element analysis

## Abstract

**Objectives** This study aimed to assess the influence of two key design parameters on the torsional resistance of endodontic rotary files: the ratio of the equivalent radius ( *r _e_* ) to the polar moment of inertia ( *J* ), or *r _e_* / *J* ratio, and the percentage of the inner core area. Understanding these factors can guide the development of files with improved performance during root canal procedures.

**Materials and Methods** Finite element analysis was employed to simulate the behavior of rotary files under torsional loading conditions. This method allowed for the investigation of maximum shear stress across various cross-sections (D _4_ –D _16_ ) of the files. The relationship between the *r _e_* / *J* ratio and the maximum shear stress was also evaluated. To assess the impact of cross-sectional design modifications on stress distribution, the study analyzed files with progressively changing configurations.

**Results** Regions situated outside the inner core circle experienced lower shear stress compared with a circular shaft. Furthermore, a strong linear correlation was observed between the maximum shear stress experienced by the file, the applied torque during operation, and the *r _e_* / *J* ratio. Significantly, the study established a connection between the percentage of the inner core area and the torsional resistance of the file. Files with a larger inner core area exhibited a lower coefficient ( *C* ) within a newly derived torsional formula. This lower *C* value directly translated to a reduction in the maximum shear stress experienced by the file. In essence, files with a higher percentage of inner core area demonstrated enhanced torsional resistance, allowing them to withstand higher torsional loads encountered during root canal procedures.

**Conclusion** This study identified the *r _e_* / *J* ratio and the percentage of inner core area as the most critical design factors influencing the torsional resistance of rotary files. Files with a lower *r _e_* / *J* ratio and a larger inner core area experienced lower shear stress, resulting in enhanced torsional resistance and potentially reducing the risk of torsional fracture during use. These findings offer valuable insights for both clinicians selecting rotary files and manufacturers designing future iterations, ultimately contributing to improved safety and efficacy during root canal treatments.

## Introduction

Nickel–titanium (NiTi) rotary files have revolutionized endodontics due to their superior canal shaping ability and reduced risk of procedural errors during root canal preparation. 1 2 3 4 5 However, a potential complication remains; file fracture within the canal, which can obstruct cleaning and shaping and necessitate retreatment. 6 7 8 9 10 11 These fractures can be categorized as flexural fatigue or torsional fracture. 12 13 Torsional fracture occurs when a file encounters significant resistance from dentine walls under continuous torque from the motor. This scenario is particularly likely when the file tip inadvertently engages the dentine wall, leading to a build-up of torsional shear stress. Extensive *in vitro* research has investigated the mechanical properties of rotary files, focusing on both flexural strength and torsional resistance. 14 15 16 17 18 19 20 21 Finite element analysis (FEA) has also been employed in some studies. 22 23 24 However, most of these studies used commercially available rotary files as test samples and reported results in terms of the files' overall strength, which encompasses various file parameters such as material, cross-sectional configuration, taper, size, and so forth. 25 26 Consequently, the specific influence of each parameter remains inconclusive.

Prior research has explored various factors influencing the torsional resistance of rotary files. Seracchiani et al 27 found that flexural stress positively influences resistance to torsional failure, but another study reported conflicting results. 28 Additionally, the motion of the motor (rotary, reciprocating, or axial) 29 30 and instrument length 31 can influence the generated torsional stress. While shear stress distribution appears independent of cross-sectional eccentricity, 32 33 the overall cross-sectional configuration significantly impacts stress distribution, flexibility, and ultimately, torsional resistance. 34 35 36 Strategies to improve torsional resistance include reducing pitch and increasing cross-sectional area. 37 Additionally, Zanza et al 38 recently identified the polar moment of inertia as the most critical cross-sectional parameter governing torsional resistance. The polar moment of inertia ( *J* ) reflects a cross-section's ability to resist twisting. It is a crucial parameter used to calculate stress and angular displacement in shafts under torque. Measured in units of length to the fourth power, *J* is calculated by integrating the product of a tiny area element and its squared distance from the shaft's center axis. For rotary files with similar shapes, a larger cross-sectional area translates to a higher *J* value. Conversely, among files with equal areas, elements positioned further from the axis contribute to a greater *J* .

The finding by Zanza et al 38 corresponded with the analytical solution for the torsion of a circular shaft problem. 39 The maximum shear stress experienced by a circular shaft under torsion is described by the well-established torsional formula:



where *τ* max is the maximum torsional shear stress on a given cross-section, *T* is the applied torque, *J* is the polar moment of inertia of the cross-sectional area, which can be determined from  , and *r* is the radius of the shaft. Consistent with the established principle of linear shear stress distribution in circular shafts under torsion, this study hypothesizes that the maximum shear stress in a rotary file subjected to torsional load is governed by two key factors: applied torque ( *T* ) and the ratio of equivalent radius ( *r _e_* ) to the polar moment of inertia ( *J* ) of the cross-section ( *r _e_* / *J* ). The equivalent radius represents the radius of a hypothetical circle with an area equal to the file's cross-section. Additionally, the inner core diameter has also been recognized as a factor influencing the torsional resistance of rotary files. 29 It is well known that a larger cross-sectional area enhances torsional resistance in rotary instruments. 40 This study aims to investigate the impact of modifying the inner core diameter on a file' s torsional resistance while maintaining a constant basic cross-sectional shape.

While prior research has explored the influence of design on torsional resistance in rotary files, a comprehensive scientific understanding based on numerical analysis remains limited. This study addresses this gap by employing FEA to evaluate the torsional shear stress distribution within the files. Specifically, we investigated the correlation between the cross-sectional *r _e_* / *J* ratio and the maximum-induced shear stress. Additionally, the impact of modifying the inner core diameter on the torsional behavior was examined while maintaining the overall basic cross-sectional shape.

## Materials and Methods

This study employed the finite element method to model and evaluate the torsional shear stress distribution in endodontic files with four distinct cross-sectional geometries: triangular (tri), square (sq), rectangular (rec), and S-shape (S). The models were subjected to torsional loading following ISO 3630–1 guideline. 41 We investigated the influence of three key parameters on the maximum shear stress: the ratio of equivalent radius to polar moment of inertia ( *r _e_* / *J* ), applied torque, and the ratio of inner core area to total cross-sectional area. Furthermore, an empirical formula for maximum shear stress was derived, building upon the established torsional formula for circular shafts.

### Finite Element Modeling and Stress Analysis

**Fig. 1:**
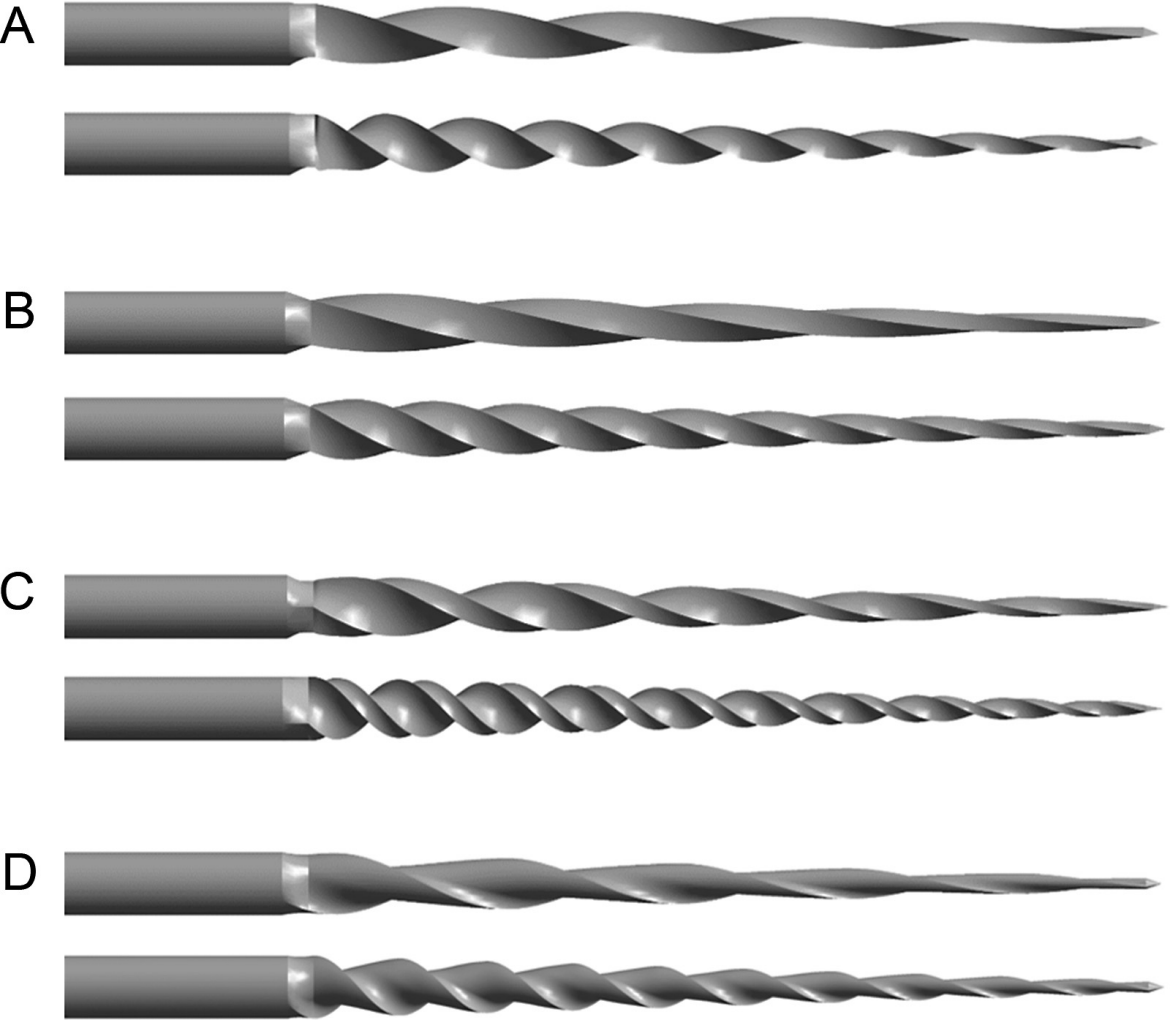
Endodontic rotary files with (A) triangular, (B) square, (C) rectangular, and (D) S-shaped cross-sectional designs used in this study. Both 5- and 10-thread files were included in this study.

**Fig. 2:**
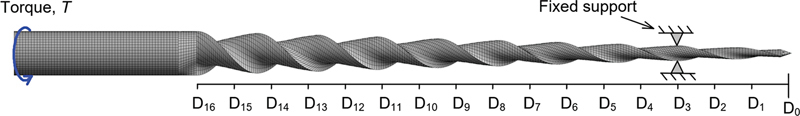
The finite element model of the 10-thread S-shaped cross-sectional file with a fixed support at D _3_ and applied torque, *T* , at the shaft of the file.

Solid models of the rotary files were generated using computer-aided design (CAD) software (SolidWorks, Dassault Systèmes, Waltham, Massachusetts, United States). Four experimental groups were established according to their cross-sectional configurations: tri, sq, rec, and S. Within each group, four specimens were analyzed with tapers of 4 or 6% and threads of 5 or 10 along a 16-mm active section. A file with a tri cross-section, 4% taper, and five threads was designated as “tri-4%-5.” All files had a consistent outer diameter of 0.25 mm at cross-section D _0_ and were fabricated from NiTi alloy with identical mechanical properties ( *E* = 36 GPa, ν = 0.33). 38 Fig. 1 presents representative three-dimensional (3D) solid models (6% taper, 5 and 10 threads) for each of the four cross-sectional designs. The tri design has an equilateral cross-section, and the rec design has an aspect ratio of 2:1. These models were then meshed for FEA using ANSYS 2022 R2 software (ANSYS Inc., Canonsburg, Pennsylvania, United States), as shown in Fig. 2 . The meshed model was fixed at cross-section D _3_ (3 mm from the tip), while the applied torque ( *T* ) was loaded onto the file shaft. The positions of cross-sections D _0_ –D _16_ are also presented in Fig. 2 . The software facilitated static analysis to determine the torsional shear stress distribution. High-order, 3D, 20-node solid hexahedral elements (SOLID186) were used. A mesh convergence study ensured solution accuracy by analyzing models with varying element sizes. Convergence was achieved when changes in maximum shear stress became negligible and stress distribution contours remained consistent.

### Relationship between the Ratio of *r _e_* / *J* and Maximum Shear Stress

Following the generation of finite element models for the rotary files, we investigated the correlation between the maximum shear stress and the *r _e_* / *J* ratio across various cross-sections. The models were subjected to a standardized torsional load of 2.5 N-mm applied to the file shaft, with a fixed boundary condition at cross-section D _3_ . FEA enabled us to identify the peak torsional shear stresses across sections D _4_ –D _16_ . We then conducted a detailed examination of the stress distribution within each of these sections. The equivalent radius ( *r _e_* ) and polar moment of inertia ( *J* ) for each cross-section were calculated based on the corresponding area obtained from the CAD software. Subsequently, the *r _e_* / *J* ratio was determined for each section. Finally, we plotted the maximum shear stress values against the corresponding *r _e_* / *J* ratios to visualize the relationship between these parameters.

### Relationship between Applied Torque and Maximum Shear Stress

This section explored the influence of applied torque on maximum shear stress across the files' cross-sections. Standardized rotary files with all four geometries, a 6% taper, and five threads were included in this simulation. The models were subjected to identical support and loading conditions as in the previous section, with varying torque levels ranging from 1 to 5 N-mm. We then plotted the maximum shear stress observed at cross-section D _8_ for each geometry as a function of the applied torque. Building upon the established relationships between maximum shear stress and both the *r _e_* / *J* ratio (previously analyzed) and applied torque (investigated in this section), a torsional formula specific to rotary files was subsequently derived. The coefficients within this formula were determined based on the slopes of the linear relationships observed in the aforementioned analyses.

### Relationship between the Percentage of Inner Core Area and Maximum Shear Stress

**Fig. 3:**
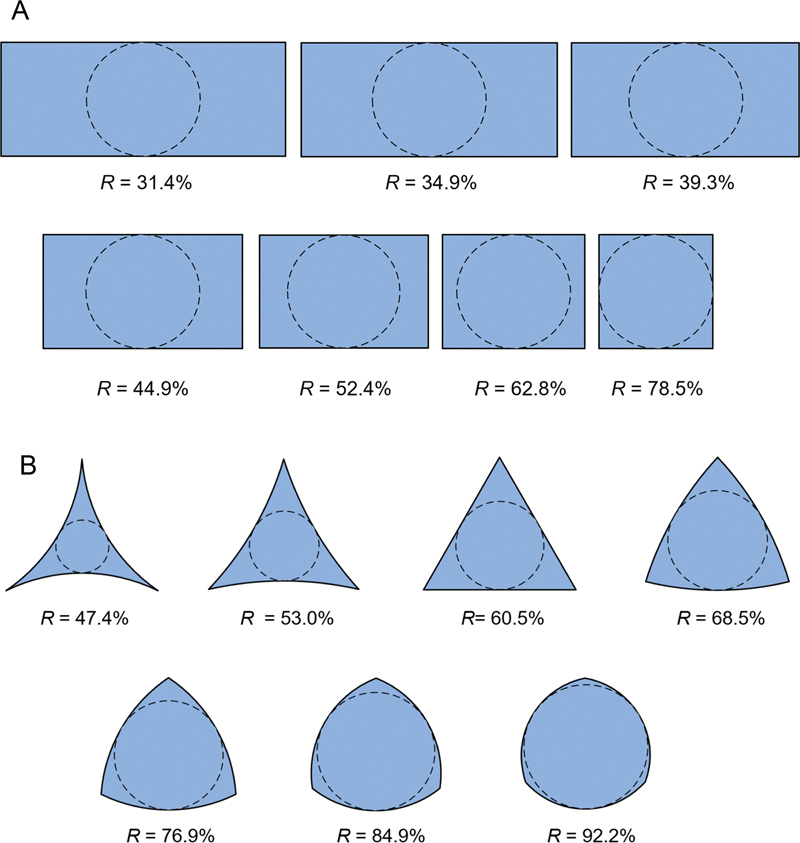
The cross-sectional configuration of the (A) rectangular and (B) triangular-based cross-sectional designs, along with the percentage of the inner core area ( *R* ). The cross-sectional configuration of the files was gradually varied so that the effect of the percentage of the inner core area on the maximum shear stress was investigated.

The final investigation explored the influence of the ratio of inner core area to the total cross-sectional area, referred to as the percentage of inner core area ( *R* ), on the maximum observed shear stress. This analysis focused on two sets of files: rec and tri-based cross-sections. During model creation, the software automatically calculated the cross-sectional area and inner core area (represented by a circle) for each file. The percentage of inner core area ( *R* ) was then derived from these values. For this investigation, the cross-sectional geometry of each design was systematically modified to create test specimens with varying *R* values, as illustrated in Fig. 3 . The figure also illustrates the inner core circle and corresponding *R* value for each design. Seven rec-based designs with aspect ratios (width per height) varying from 2.5 to 1 were included ( Fig. 3A ). A similar number of tri-based designs were investigated ( Fig. 3B ), including both straight-sided and curved-sided triangles. In these designs, *R* was adjusted by modifying the triangle's side configuration. FEA was employed to determine the maximum torsional shear stress induced across cross-sections D _4_ –D _16_ for files with varying inner core area ratios ( *R* ). Based on the maximum shear stress, an empirical formula for the maximum torsional shear stress specific to these rotary files was developed. This formula shares similarities with the classical torsional formula for circular shafts ( Equation 1 ) and will be subsequently compared.

## Results

A mesh convergence study was performed to ensure the accuracy of the finite element solutions used in subsequent analyses. Table 1 summarizes the element and node counts for each FEA model. Most models employed an element size of 0.03 mm. However, models with a rec cross-section and 4% taper required a finer element size of 0.02 mm, while models with a tri cross-section and 6% taper could utilize a larger element size of 0.04 mm without compromising accuracy.

**Table 1:** Number of elements and nodes in the finite element model of files with various configurations

File configuration	Number of elements	Number of nodes	Element size (mm)
tri − 4% − 5	129,472	545,835	0.03
tri − 4% − 10	151,488	630,384	
tri − 6% − 5	88,382	369,889	0.04
tri − 6% − 10	101,474	419,614	
sq − 4% − 5	54,000	249,821	0.03
sq − 4% − 10	55,600	257,213	
sq − 6% − 5	65,824	300,696	0.03
sq − 6% − 10	69,091	315,600	
rec − 4% − 5	166,800	744,589	0.02
rec − 4% − 10	184,800	824,869	
rec − 6% − 5	55,958	261,284	0.03
rec − 6% − 10	65,366	305,156	
S − 4% − 5	121,426	514,241	0.03
S − 4% − 10	114,145	489,871	
S − 6% − 5	278,077	1,137,207	0.03
S − 6% − 10	336,996	1,364,835	

Abbreviations: rec, rectangle; S, S-shape; sq, square; tri, triangular.

**Fig. 4:**
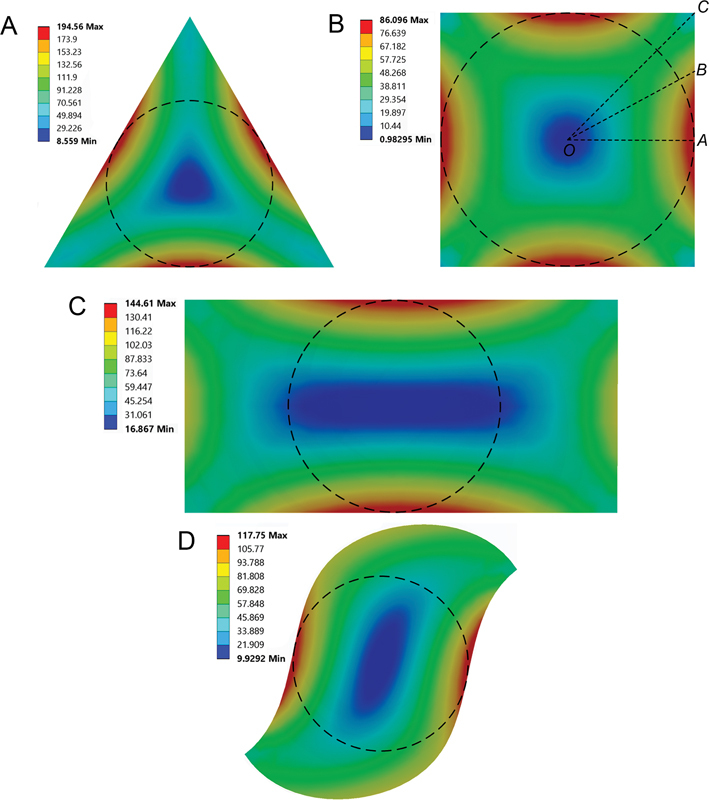
Distribution of torsional shear stress on the cross-section D _8_ of the five threads and 6% taper files with (A) triangular, (B) square, (C) rectangular, and (D) S-shaped cross-sectional designs under an applied torque of 2.5 N-mm.

**Fig. 5:**
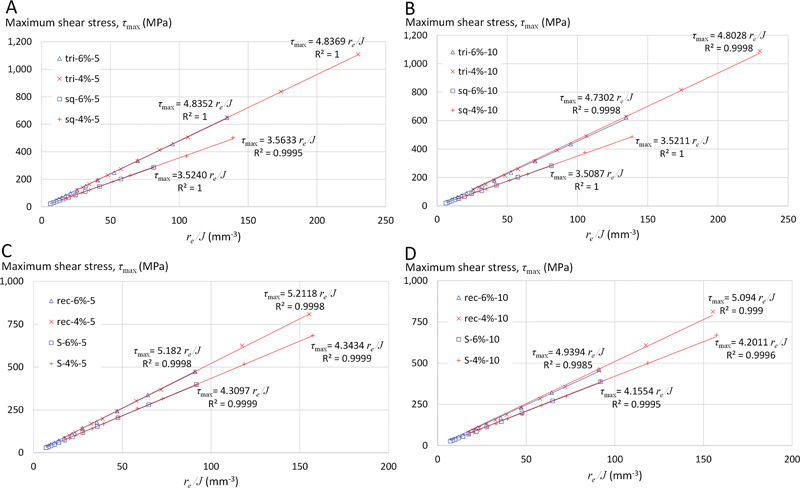
Plots of maximum shear stress at cross-sections D _4_ –D _16_ along the axis of the file versus *r _e_* / *J* ratio: (A) 5-thread tri and sq cross-sectional files; (B) 10-thread tri and sq cross-sectional files; (C) 5-thread rec and S cross-sectional files; and (D) 10-thread rec and S cross-sectional files. rec, rectangle; S, S-shape; sq, square; tri, triangular

For the first part of the study, Fig. 4 presents the distribution of shear stress across cross-section D _8_ for five threads, 6% taper files with all four geometries under a 2.5 N-mm applied torque. Contour plots visualize the distribution, enabling the identification of maximum shear stress values within each cross-section. Similar stress patterns were observed in other sections of this file and files with different thread counts or taper angles. The dashed circle in the plots represents the inner core circle. As expected, torsional shear stress is zero at the file axis and reaches its peak where the inner core circle meets the cross-sectional perimeter. Fig. 5 presents the relationship between maximum shear stress (across D _4_ –D _16_ ) and the *r _e_* / *J* ratio for all geometries. A linear relationship was observed, prompting linear regression analysis to quantify the relationship. The equations and corresponding coefficients of determination ( *R* ^2^ ) are displayed on each plot.

**Fig. 6:**
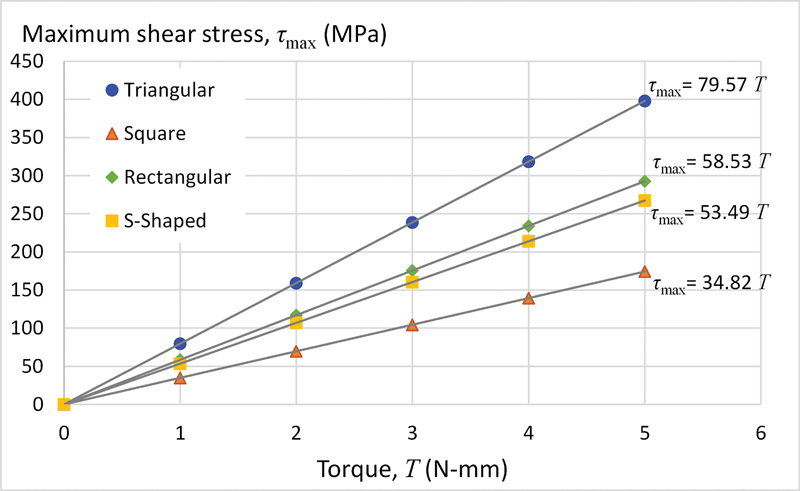
Plots of the maximum shear stress on cross-section D _8_ versus applied torque, *T* .

For the second part of the study, Fig. 6 presents the relationship between maximum shear stress on cross-section D _8_ and applied torque. A linear trend is observed, with the tri design exhibiting a steeper slope compared with the rec, S, and sq designs, respectively. Combining these results with those from the previous section, we conclude that maximum shear stress varies linearly with both applied torque ( *T* ) and the *r _e_* / *J* ratio. This relationship can be expressed mathematically as:



where *C* is the coefficient determined from the slope of the plots in Fig. 5 . The coefficients *C* for each file configuration are presented in Table 2 .

**Table 2:** Coefficients *C* in the maximum shear stress formula for various cross-sectional configurations

Cross-sectional configuration	Taper	Coefficients *C*	
		5 Threads	10 Threads
Triangular	4%	1.935	1.921
	6%	1.934	1.892
Square	4%	1.425	1.408
	6%	1.410	1.403
Rectangular	4%	2.085	2.038
	6%	2.073	1.976
S-shaped	4%	1.737	1.680
	6%	1.724	1.662

**Fig. 7:**
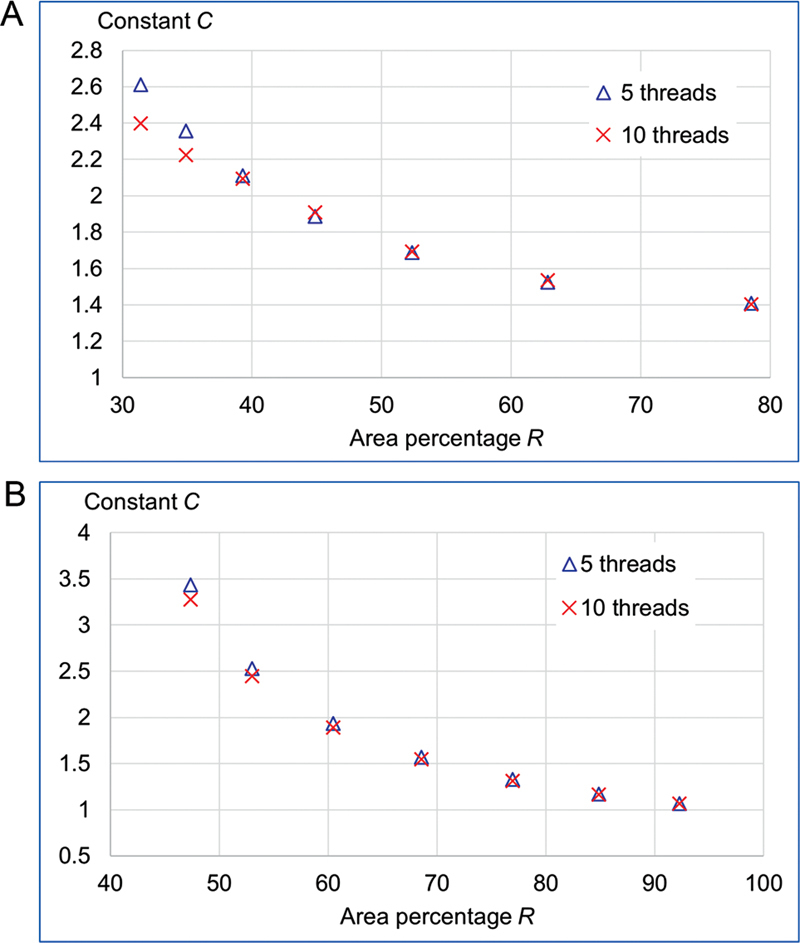
Relationship between the constant *C* and percentage of the inner core area ( *R* ) of the files with (A) rectangular cross-section and (B) concave and convex triangular cross-sections.

The final investigation explored the influence of the inner core area ratio ( *R* ) on maximum shear stress. The coefficients *C* in the torsion formula ( Equation 2 ) for files with rec and tri-based cross-sections were plotted against the percentage of inner core area *R* in Fig. 7 . The coefficient *C* was chosen as a parameter to represent the torsional resistance of the files because it indicates the maximum shear stress value for a given applied torque and *r _e_* / *J* ratio. For both cross-sectional configurations, coefficient *C* decreases with an increasing percentage of the inner core area.

## Discussion

Failure of NiTi rotary files during root canal procedures occurs due to cyclic fatigue or excessive torsional loads. The file's torsional resistance, directly related to the shear stress it experiences during operation, has been investigated in previous studies focusing on various geometric design parameters. 37 42 In the context of rotary files operating in root canals, von Mises stress and maximum principal stress are commonly used failure parameters. When subjected to torsional loading, shear stress predominates and exhibits a direct correlation with von Mises stress and maximum principal stress. Consequently, this study centered on shear stress and its empirical formula for maximum shear stress. The polar moment of inertia of the cross-sectional area was identified as the most important parameter over mass and cross-sectional area. 38 The utilization of the finite element method in this study presents a distinct advantage over experimental approaches, as it allows for precise design and control of these parameters. The first part of the study focused on shear stress distribution across different rotary file cross-sections, as shown in Fig. 4 . The highest stress was observed at the inner core–outer surface interface. Interestingly, regions outside the inner core (e.g., corners of tri or sq cross-sections, S tips) experienced lower stress despite being farther from the center, unlike a circular shaft where stress increases with distance from the axis. This highlights the unique stress distribution in noncircular cross-sections, where specific regions experience reduced shear forces despite being farther from the axis. To illustrate this, we examined the sq cross-section presented in Fig. 4B and its stress distribution along lines *OA* , *OB* , and *OC* . Unlike a circular shaft with linear stress distribution (zero at the axis), stress on line *OA* (within the inner core) increases from zero to a maximum. Conversely, stress on line *OC* (partially outside the inner core) initially increases, plateaus in the middle, and then decreases. Furthermore, line *OB* (between *OA* and *OC* ) exhibits a lower rate of stress increase compared with *OA* . This demonstrates that elements along lines partially outside the inner core experience lower torsional stress. The shear stress that is not fully sustained by the initial location is redistributed to other elements within the cross-section. Consequently, the maximum shear stress in noncircular sections exceeds that of a circular shaft with equal cross-sectional area due to the presence of lower-stress regions. This observation holds true for other cross-sectional configurations.

Next, the study investigated how maximum shear stress varies across different file cross-sections (D _4_ –D _16_ ) and its relationship with the *r _e_* / *J* ratio. As shown in Fig. 5 , a strong linear correlation ( *R* ^2^ ≈ 1) was observed between these parameters. Similarly, Fig. 6 demonstrates a linear dependence of maximum shear stress on the applied torque. These findings suggest that maximum shear stress depends linearly on both the *r _e_* / *J* ratio and applied torque, as described in Equation 2 . The coefficient *C* in the equation, obtained from the slopes in Fig. 5 divided by applied torque of 2.5 N-mm, represents a file's relative susceptibility to shear stress compared with a circular shaft ( *C* = 1, Equation 1 ). In this study, *C* values ranged from 1.4 (sq) to 2.0 (rec), indicating that rec cross-sectional files are nearly twice as susceptible to shear stress as a circular shaft. The rec design exhibited the highest *C* , while the sq design had the lowest. Notably, *C* values for files with the same cross-section but varying thread count or taper percentage differed by less than 5%. The taper percentage had a negligible effect on maximum shear stress, while the number of threads slightly influenced *C* for the rec design only. In conclusion, the coefficient *C* , specific to each cross-section, allows for the prediction of maximum shear stress. The primary factor influencing stress is the cross-sectional configuration, with thread count and taper having minimal impact. The *r _e_* / *J* ratio of the cross-section of the file significantly impacts the magnitude of shear stress experienced under torsional load.

The final investigation of this study focused on the impact of cross-sectional design modifications on the maximum shear stress experienced within the rotary file. The coefficient *C* , derived from the established torsional formula, Equation 2 , served as a crucial parameter in this analysis. A lower *C* value directly correlates to a lower magnitude of maximum shear stress within the file. Fig. 7 effectively demonstrates the relationship between coefficient *C* and the percentage of inner core area for both rec and tri-based cross-sectional configurations. For example, in Fig. 7A , a rec file with five threads exhibits a significant decrease in coefficient *C* , from 2.6 (low inner core area) to 1.4 (high inner core area) as the percentage of inner core area increases. This observation aligns with the previously discussed shear stress distribution across the cross-section, as illustrated in Fig. 4 . Notably, shear stress tends to increase at a slower rate, or even decrease, in regions outside the inner core circle compared with areas situated within it. In a circular shaft, the inner core circle and the perimeter of the shaft are identical, resulting in shear stress linearly varying with the distance from the axis in all directions. However, for a noncircular cross-section like that of a rotary file, there are portions of the cross-section outside the inner core circle that sustain lower shear stress. Consequently, a portion of the cross-section inside the inner core circle bears the remaining internal force, leading to a higher maximum shear stress on that part. Therefore, a file with a higher percentage of inner core area has a smaller coefficient *C* and consequently experiences lower maximum shear stress. This cross-sectional design exhibits higher torsional resistance, allowing it to withstand higher torque, compared with designs with a lower percentage of inner core area. This finding can be applied to the design of file cross-sections to achieve optimal torsional resistance. To enhance torsional resistance, a file should have a lower percentage of area outside the inner core circle. For example, a tri-based cross-sectional file with convex sides can endure higher torsional loads than those with concave sides. Similarly, a sq cross-sectional design is preferred over a rec one, and an S cross-sectional design with an aspect ratio close to 1 is favored over designs with higher aspect ratios.

Expanding on prior research 38 that identified the polar moment of inertia as a key factor in torsional resistance, this study demonstrates that torsional shear stress can be described by the proposed torsional formula ( Equation 2 ). This formula offers a valuable tool for designing rotary files with various cross-sectional configurations.

## Conclusion

This study revealed a linear relationship between maximum shear stress, applied torque, and the *r _e_* / *J* ratio across various rotary file cross-sections. Furthermore, an empirically derived coefficient ( *C* ) from FEA accounts for the influence of cross-sectional design on stress ( Equation 2 ). Notably, files with a larger percentage of inner core area exhibit a lower *C* and consequently experience lower shear stress and enhanced torsional resistance. Consequently, a rotary file with a reduced *r _e_* / *J* ratio and an increased percentage of the inner core area demonstrate enhanced torsional resistance. These findings offer valuable guidance for both clinicians and manufacturers. Clinicians can leverage this knowledge to select files with minimal risk of torsional fractures, while manufacturers can utilize it to design files with improved resistance. However, a comprehensive evaluation should consider additional factors such as bending stress, fatigue, flexibility, and cutting efficiency.

## References

[1] Esposito P T Cunningham C J A comparison of canal preparation with nickel-titanium and stainless steel instruments J Endod 1995 21 04 173 176 7673815 10.1016/S0099-2399(06)80560-1

[2] Schäfer E Schlingemann R Efficiency of rotary nickel-titanium K3 instruments compared with stainless steel hand K-Flexofile. Part 2. Cleaning effectiveness and shaping ability in severely curved root canals of extracted teeth Int Endod J 2003 36 03 208 217 12657147 10.1046/j.1365-2591.2003.00644.x

[3] Nakatsukasa T Ebihara A Kimura S Comparative evaluation of mechanical properties and shaping performance of heat-treated nickel titanium rotary instruments used in the single-length technique Dent Mater J 2021 40 03 743 749 33518688 10.4012/dmj.2020-255

[4] Lakshmanan L Jeevanandan G Maganur P C Vishwanathaiah S Fracture incidence of Kedo-S square pediatric rotary files: a prospective clinical study Eur J Dent 2022 16 03 594 598 34863082 10.1055/s-0041-1735935 PMC9507586

[5] Campos G O Fontana C E Vieira V TL Elias C N de Martin A S Bueno C EDS Influence of heat treatment of nickel-titanium instruments on cyclic fatigue resistance in simulated curved canals Eur J Dent 2023 17 02 472 477 36195211 10.1055/s-0042-1747952 PMC10329553

[6] Cheung G SP Instrument fracture: mechanisms, removal of fragments, and clinical outcomes Endod Topics 2007 16 01 1 26

[7] McGuigan M B Louca C Duncan H F Endodontic instrument fracture: causes and prevention Br Dent J 2013 214 07 341 348 23579132 10.1038/sj.bdj.2013.324

[8] Madarati A A Hunter M J Dummer P M Management of intracanal separated instruments J Endod 2013 39 05 569 581 23611371 10.1016/j.joen.2012.12.033

[9] Sekar V Kumar R Nandini S Ballal S Velmurugan N Assessment of the role of cross section on fatigue resistance of rotary files when used in reciprocation Eur J Dent 2016 10 04 541 545 28042272 10.4103/1305-7456.195171 PMC5166313

[10] Pinto J C Torres F FE Santos-Junior A O Duarte M AH Guerreiro-Tanomaru J M Tanomaru-Filho M Safety and effectiveness of additional apical preparation using a rotary heat-treated nickel-titanium file with larger diameter and minimum taper in retreatment of curved root canals Eur J Dent 2021 15 02 247 252 33622011 10.1055/s-0041-1723065 PMC8184271

[11] Akçay A Gorduysus M Gorduysus M O Annamma L M Müftüoglu S A comparative evaluation of the cleaning efficacy of five different root canal irrigation devices: a histological study Eur J Dent 2024 18 03 827 833 37995725 10.1055/s-0043-1774325 PMC11290919

[12] Parashos P Gordon I Messer H H Factors influencing defects of rotary nickel-titanium endodontic instruments after clinical use J Endod 2004 30 10 722 725 15448468 10.1097/01.don.0000129963.42882.c9

[13] Sattapan B Nervo G J Palamara J E Messer H H Defects in rotary nickel-titanium files after clinical use J Endod 2000 26 03 161 165 11199711 10.1097/00004770-200003000-00008

[14] Varghese N O Pillai R Sujathen U N Sainudeen S Antony A Paul S Resistance to torsional failure and cyclic fatigue resistance of ProTaper Next, WaveOne, and Mtwo files in continuous and reciprocating motion: An in vitro study J Conserv Dent 2016 19 03 225 230 27217634 10.4103/0972-0707.181937 PMC4872575

[15] Pedullà E Lo Savio F Boninelli S Torsional and cyclic fatigue resistance of a new nickel-titanium instrument manufactured by electrical discharge machining J Endod 2016 42 01 156 159 26586518 10.1016/j.joen.2015.10.004

[16] Elsaka S E Elnaghy A M Badr A E Torsional and bending resistance of WaveOne Gold, Reciproc and Twisted File Adaptive instruments Int Endod J 2017 50 11 1077 1083 27917513 10.1111/iej.12728

[17] Silva E Oliveira de Lima C Vieira V Antunes H Lima Moreira E J Versiani M Cyclic fatigue and torsional resistance of four martensite-based nickel titanium reciprocating instruments Eur Endod J 2020 5 03 231 235 33353907 10.14744/eej.2020.16878 PMC7881387

[18] Hiran-Us S Morakul S Effect of temperatures on cyclic fatigue resistance of 3 different Ni-Ti alloy files Int Dent J 2023 73 06 904 909 37423864 10.1016/j.identj.2023.06.008 PMC10658440

[19] Yum J Cheung G S Park J K Hur B Kim H C Torsional strength and toughness of nickel-titanium rotary files J Endod 2011 37 03 382 386 21329826 10.1016/j.joen.2010.11.028

[20] Alqedairi A Alfawaz H Abualjadayel B Alanazi M Alkhalifah A Jamleh A Torsional resistance of three ProTaper rotary systems BMC Oral Health 2019 19 01 124 31226984 10.1186/s12903-019-0820-7 PMC6588856

[21] Sivas Yilmaz Ö Keskin C Aydemir H Comparison of the torsional resistance of 4 different glide path instruments J Endod 2021 47 06 970 975 33640424 10.1016/j.joen.2021.02.009

[22] Necchi S Petrini L Taschieri S Migliavacca F A comparative computational analysis of the mechanical behavior of two nickel-titanium rotary endodontic instruments J Endod 2010 36 08 1380 1384 20647101 10.1016/j.joen.2010.03.026

[23] Arbab-Chirani R Chevalier V Arbab-Chirani S Calloch S Comparative analysis of torsional and bending behavior through finite-element models of 5 Ni-Ti endodontic instruments Oral Surg Oral Med Oral Pathol Oral Radiol Endod 2011 111 01 115 121 21176826 10.1016/j.tripleo.2010.07.017

[24] Martins S CS Silva J D Garcia P R Viana A CD Buono V TL Santos L A Influence of cyclic loading in NiTi austenitic and R-phase endodontic files from a finite element perspective Clin Oral Investig 2022 26 05 3939 3947 10.1007/s00784-021-04360-2 35039941

[25] Martins J NR Silva E JNL Marques D Comparison of five rotary systems regarding design, metallurgy, mechanical performance, and canal preparation-a multimethod research Clin Oral Investig 2022 26 03 3299 3310 10.1007/s00784-021-04311-x 34860307

[26] Kaval M E Capar I D Ertas H Sen B H Comparative evaluation of cyclic fatigue resistance of four different nickel-titanium rotary files with different cross-sectional designs and alloy properties Clin Oral Investig 2017 21 05 1527 1530 10.1007/s00784-016-1917-x 27456784

[27] Seracchiani M Miccoli G Di Nardo D Effect of flexural stress on torsional resistance of NiTi instruments J Endod 2021 47 03 472 476 33096192 10.1016/j.joen.2020.10.011

[28] Jamleh A Almedlej R Alomar R Almayouf N Alfadley A Alfouzan K Evidence for reduced torsional resistance of rotary files under curved position Saudi Dent J 2021 33 07 614 619 34803309 10.1016/j.sdentj.2020.07.005 PMC8589599

[29] Liang Y Yue L Evolution and development: engine-driven endodontic rotary nickel-titanium instruments Int J Oral Sci 2022 14 01 12 35181648 10.1038/s41368-021-00154-0 PMC8857196

[30] Lee J Y Kwak S W Ha J H Kim H C Ex-vivo comparison of torsional stress on nickel-titanium instruments activated by continuous rotation or adaptive motion Materials (Basel) 2020 13 08 1900 32316559 10.3390/ma13081900 PMC7215760

[31] Gambarini G Seracchiani M Zanza A Miccoli G Del Giudice A Testarelli L Influence of shaft length on torsional behavior of endodontic nickel-titanium instruments Odontology 2021 109 03 568 573 33245455 10.1007/s10266-020-00572-2 PMC8178130

[32] Ha J H Kwak S W Versluis A Lee C J Park S H Kim H C The geometric effect of an off-centered cross-section on nickel-titanium rotary instruments: a finite element analysis study J Dent Sci 2017 12 02 173 178 30895044 10.1016/j.jds.2016.11.005 PMC6395356

[33] Martins S CS Garcia P R Viana A CD Buono V TL Santos L A Off-centered geometry and influence on NiTi endodontic File performance evaluated by finite element analysis J Mater Eng Perform 2020 29 04 2095 2102

[34] Xu X Eng M Zheng Y Eng D Comparative study of torsional and bending properties for six models of nickel-titanium root canal instruments with different cross-sections J Endod 2006 32 04 372 375 16554216 10.1016/j.joen.2005.08.012

[35] Elnaghy A M Elsaka S E Torsion and bending properties of OneShape and WaveOne instruments J Endod 2015 41 04 544 547 25534072 10.1016/j.joen.2014.11.010

[36] Kim H C Kim H J Lee C J Kim B M Park J K Versluis A Mechanical response of nickel-titanium instruments with different cross-sectional designs during shaping of simulated curved canals Int Endod J 2009 42 07 593 602 19467053 10.1111/j.1365-2591.2009.01553.x

[37] Baek S-H Lee C-J Versluis A Kim B-M Lee W Kim H-C Comparison of torsional stiffness of nickel-titanium rotary files with different geometric characteristics J Endod 2011 37 09 1283 1286 21846549 10.1016/j.joen.2011.05.032

[38] Zanza A Seracchiani M Di Nardo D Reda R Gambarini G Testarelli L A paradigm shift for torsional stiffness of nickel-titanium rotary instruments: a finite element analysis J Endod 2021 47 07 1149 1156 33915175 10.1016/j.joen.2021.04.017

[39] Hibbeler R C Mechanics of Materials Harlow, United Kingdom Pearson 2018

[40] El-Anwar M I Yousief S A Kataia E M El-Wahab T M Finite element study on continuous rotating versus reciprocating nickel-titanium instruments Braz Dent J 2016 27 04 436 441 27652707 10.1590/0103-6440201600480

[41] International Organization for Standardization. ISO 3630-1: Dental root canal instruments-Part 1: files, reamers, barbed broaches, rasps, paste carriers, explorers and cotton broaches. Geneva, Switzerland: International Organization for Standardization;1992

[42] He R Ni J Design improvement and failure reduction of endodontic files through finite element analysis: application to V-Taper file designs J Endod 2010 36 09 1552 1557 20728726 10.1016/j.joen.2010.06.002

